# Epidemiology, pathogenesis, and diagnosis of Aleutian disease caused by Aleutian mink disease virus: A literature review with a perspective of genomic breeding for disease control in American mink (*Neogale vison*)

**DOI:** 10.1016/j.virusres.2023.199208

**Published:** 2023-08-28

**Authors:** Seyed Milad Vahedi, Siavash Salek Ardestani, Mohammad Hossein Banabazi, Fraser Clark

**Affiliations:** a Department of Animal Science and Aquaculture, Dalhousie University, Bible Hill, NS B2N5E3, Canada; b Department of Animal Science, University of Zanjan, Zanjan 4537138791, Iran; c Department of animal breeding and genetics (HGEN), Centre for Veterinary Medicine and Animal Science (VHC), Swedish University of Agricultural Sciences (SLU), Uppsala 75007, Sweden; d Department of Biotechnology, Animal Science Research Institute of IRAN (ASRI), Agricultural Research, Education & Extension Organization (AREEO), Karaj 3146618361, Iran

**Keywords:** Aleutian disease, Aleutian mink disease virus, American mink, Neogale vison, Control, Genomic selection

## Abstract

Aleutian disease (AD) is a multi-systemic infectious disease in American mink (*Neogale vison*) caused by the Aleutian mink disease virus (AMDV). Commonly referred to as mink plasmacytosis, AD is an economically significant disease in mink-breeding countries. Aleutian disease mainly induces weight loss, lower fertility, and dropped pelt quality in adults and can result in acute interstitial pneumonia with high mortality rates in kits. In this review, we employed the scientific literature on AD over the last 70 years to discuss the historical and contemporary status of AD outbreaks and seroprevalence in mink farming countries. We also explained different forms of AD and the differences between the pathogenicity of the virus in kits and adults. The application of the available AD serological tests in AD control strategies was argued. We explained how selection programs could help AD control and proposed different approaches to selecting animals for building AD-tolerant herds. The advantages of genomic selection for AD tolerance over traditional breeding strategies were discussed in detail. We also explained how genomic selection could help AD control by selecting tolerant animals for the next generation based on genome-wide single nucleotide polymorphisms (SNP) data and the challenges of implementing genomic selection for AD tolerance in the mink industry. This review collected the information required for designing successful breeding programs for AD tolerance. Examples of the application of information are presented, and data gaps are highlighted. We showed that AD tolerance is necessary to be among the traits that animals are selected for in the mink industry.

## Highlights

• Aleutian disease is a multi-systemic infectious disease caused by Aleutian mink disease virus.
• The infection in farmed mink is distributed worldwide, mainly among mink-breeding countries.
• Pathogenesis and clinical manifestation of infection are different in adults and kits.
• High-throughput quantitative ELISAs can monitor disease progression in positive farms.
• Genomic breeding for disease control is feasible with host SNP data and antibody-level records.

## 1 Introduction

Aleutian disease (AD), or mink plasmacytosis, is a chronic persistent infection in American mink (*Neogale vison*) caused by Aleutian mink disease virus (AMDV), which belongs to the *Carnivore amdoparvovirus 1* species within the genus *Amdoparvovirus* (Bloom et al., 1994; Canuti et al., 2015). Nearly 80 years after the first observation of AD in US mink farms, the disease is still a significant problem of the mink industry worldwide (Kowalczyk et al., 2019; Tong et al., 2020; Virtanen et al., 2019). The most common approach applied in AD-affected countries is a test-and-removal strategy; however, eradication of AD from mink ranches has not been possible in most cases. A large number of seropositive animals, and outbreaks in mink-producing countries such as Canada, Denmark, China, Russia, and Finland, suggest that the current AD control strategies lack the potency to eliminate the disease (Kashtanov and Salnikova, 2018; Knuuttila et al., 2009b; Li et al., 2012).

Experimental attempts to develop effective protective protein or deoxyribonucleic acid (DNA)-based vaccines have failed, and only partial protections have been achieved (Aasted et al., 1998; Castelruiz et al., 2005; Liu et al., 2017). Furthermore, no efficient practical treatment has been found for the disease. Given the difficulties in eradicating AD and the lack of either vaccine or therapy, an additional or complementary control strategy should be considered. One approach could be to utilize the host genetic variation in response to AD, particularly tolerant animals; for example, a similar strategy was applied for other livestock diseases such as bovine tuberculosis (Raphaka et al., 2018) or salmon infectious pancreatic necrosis (Bishop and Woolliams, 2014). Individual differential susceptibility to specific AMDV strains has been characterized, suggesting possible genetic variations among these animals in response to the virus. This highlights the potential for employing genomic tools to identify and select less susceptible animals in disease control programs.

This paper reviews the history of AD outbreaks, AMDV pathogenesis, and host-virus interaction. We also provide detailed information on how tolerant mink could be recognized and selected to improve AD immune response and control disease using currently available serological and molecular diagnostic tests. Finally, key challenges and future research opportunities for incorporating genomic selection approaches in AD control programs aiming to enhance the genetic merit of disease tolerance are described.

## 2 AD epidemiology and outbreaks

**Fig. 1:**
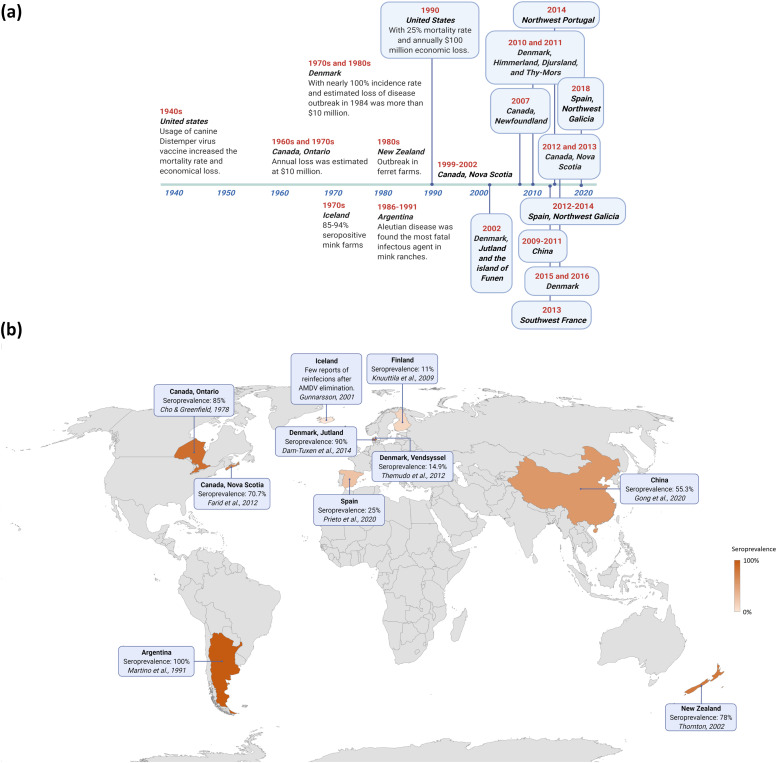
Timeline of Aleutian disease outbreaks worldwide (a) and geographical distribution of Aleutian disease seroprevalence in farmed mink (b).

AD was first described in farmed American mink in the US in the 1940s; however, the primary source of the virus by which the disease was spread among farmed animals remains unknown (Hartsough and Gorham, 1956). The majority of initial studies on AD, including the discovery of the disease, virus isolation, classification of the virus as a parvovirus, and complete genome sequencing were performed in the US (Bloom et al., 1988; Karstad and Pridham, 1962; Porter et al., 1977). Fig. 1 illustrates the timeline of AD outbreaks and the latest status of the disease seroprevalence among farmed mink worldwide. The chronological growth in the number of Aleutian disease outbreaks and involved countries could be due to the development of intensive mink breeding programs in different countries, the transport of farmed animals among nations, the pathogen exchange among farmed mink and feral animals, as well as an improvement in the diagnostic tools.

### 2.1 North America

The first detection of AD in Canada dates back to the late 1950s and is where the etiology of AD as a viral infection was first uncovered (Karstad and Pridham, 1962). Several outbreaks of AD have been reported from the 1960s and 1990s in Canada and US, centered in Ontario, Washington, and Oregon (Cho and Greenfield, 1978; Dodds and Schultz, 1998; Jackson et al., 1996; Karstad and Pridham, 1962). Following the 2000s, despite regular annual AD tests performed on the Canada's American mink populations, estimated at 1.6–1.7 million animals, Atlantic Canada has experienced several outbreaks, e.g., Nova Scotia in 1999–2002, 2012, 2013, and Newfoundland in 2007 (Canuti et al., 2016; Farid et al., 2012; Farid and Hussain, 2020; Newman and Reed, 2006). Canada's national survey, conducted in 2006 to estimate the AD prevalence in mink herds, revealed that among the 5% of seropositive ranches, 60% and 27% of them belonged to two provinces, Nova Scotia and Ontario (Newman and Reed, 2006). The high concentration of mink ranches and wild mammals harboring the AMDV made viral eradication challenging (Farid et al., 2012, 2010).

### 2.2 Europe

AD was reported in several European countries, including Czechoslovakia, Iceland, Spain, Denmark, and the Netherlands, until 2000 (Chriel, 1998; Chriél, 2000; Gunnarsson, 2001; Haagsma, 1980; Joergensen and Henriksen, 1989; Jørgensen and Bøtner, 1983; Konrád and Nevole, 1971; Larsen et al., 1984). In 2003, the first report of AD in Russia demonstrated the expansion of the virus scope to Eastern Europe (Kashtanov and Salnikova, 2018). Following the 21st century, among European countries, AD outbreaks have been frequently reported in Denmark, Spain, Portugal, and France (Hjulsager et al., 2016; Østergaard and Jensen, 2012; Prieto et al., 2020; Ryt-Hansen et al., 2017a). Although, the drop in the number of AD-positive Danish mink farms, from 100% in 1976 to 15% in 2009, and 5% in 2011, points to the success of implemented strategies in this country (Aasted et al., 1998; Themudo et al., 2011; Themudo et al., 2012; Ryt-Hansen et al., 2017b). In Denmark, special measures have been implemented, and a perimeter (eradication zone) was established around the infected regions, and the movement of animals outside of the eradication zones was restricted by legislation (Themudo et al., 2011). In another approach, test-and-removal of animals, plus stamping-out and closure of infected farms, reduced the seroprevalence of AD in Spain from 100% in 1980 to 25% in 2019 (Prieto et al., 2020).

### 2.3 Asia, Australasia, and South America

High seroprevalence of AD, up to 70%, mainly in eastern and northern China, was reported in 2000 (Gong et al., 2020). The increase in seroprevalence of disease among Chinese mink ranches from 48% (before 2010) to 61.4% (after 2010) and the disease epidemy from 2009 to 2011 among mink farms demonstrated the infection growth over time and challenges in the control of AD in this country (Gong et al., 2020; Sang et al., 2012). Moreover, an outbreak was detected in the New Zealand ferret farms in 1980s. Among ranches with clinical signs of AD, 78% were seropositive. Although no structured surveys for AD in New Zealand ferrets have been conducted, passive laboratory surveillance suggested that infection is likely to be common (Gill et al., 1988). In contrast, in Argentina, autopsies of adult mink submitted from 1986 to 1991 showed that AD was the most important cause of death and economic loss in their mink industry (Martino et al., 1991).

## 3 AMDV replication cycle, pathogenesis, and cell tropism

**Fig. 2:**
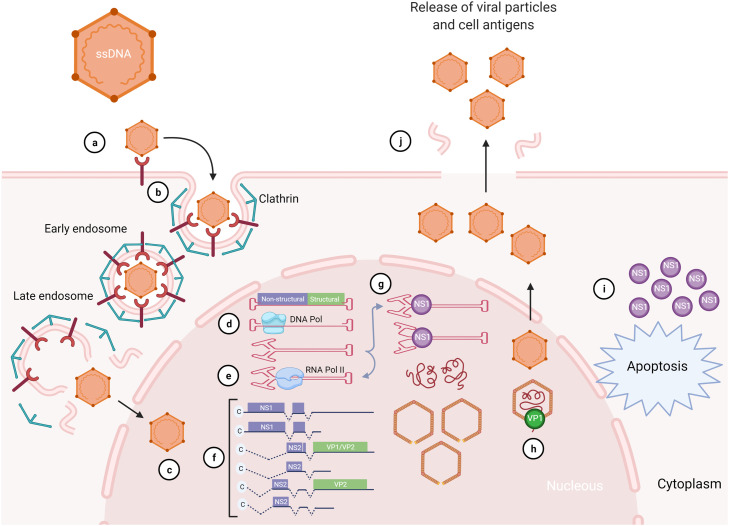
AMDV infection of mink cells (viral replication cycle). The virus replication cycle includes attachment (a), internalization (b), intracellular trafficking (c), replication (d), transcription (e and f), translation (g), encapsidation (h), apoptosis (i), and release (j).

AMDV capsid contains 4.8 kilobase pairs of single-stranded DNA (ssDNA) encoding two structural proteins, VP1 and VP2, and three nonstructural proteins, including NS1, NS2, and NS3 (Bloom et al., 1988; Li et al., 2012). VP2 is the major structural protein and the main immunogenic protein of the virus, involved in viral tropism, pathogenicity, and host selection (Clemens et al., 1992). The replication cycle of AMDV is briefly shown in Fig. 2.

When adult mink macrophages are infected by AMDV, the virus interacts with cellular Fc receptors recognizing opsonized viral particles (Dworak et al., 1997; Kanno et al., 1993). In contrast, alveolar type II cells of the lungs are mostly infected by the virus in kits (Alexandersen et al., 1987). The attachment to the host receptor initiates clathrin-mediated endocytosis of the virion into the host cell (Fig. 2a) (Parker and Parrish, 2000). Subsequent to virus-receptor interaction, AMDV is internalized into the cells by permeabilization of the host endosomal membrane (Fig. 2b) (Suikkanen et al., 2003). After several steps of intracellular trafficking in the endosome, parvoviruses are released from the endosome through a function of the PLA2 domain in VP1; however, AMDV lacks this motif, suggesting a different unknown mechanism of endosomal release from other parvoviruses (Fig. 2c) (Zádori et al., 2001). After entrance to the nucleus, the virion releases the ssDNA, which is converted to double-stranded DNA (dsDNA) using cellular DNA polymerase (DNA Pol) and other DNA replication factors (Fig. 2d) (Qiu et al., 2017). Transcription of dsDNA by ribonucleic acid polymerase II (RNA pol II) gives rise to viral pre-mRNAs when the host cell enters the S phase (Fig. 2e) (Oleksiewicz and Alexandersen, 1997). Six different mRNA will be generated from a single pre-mRNA through alternative processing of pre-mRNA (Fig. 2f) (Huang et al., 2014). AMDV probably uses rolling hairpin replication for DNA synthesis, similar to other parvoviruses (Berns, 1990). In this process, following the synthesis of dsDNA by DNA Pol, NS1 initiates the strand displacement replication, including folding and unfolding of DNA molecule repeatedly rearranged into intermediate replication form (Fig. 2g). Eventually, VP1 binds DNA while folded into a secondary intermediate replication form, resulting in the segregation and encapsidation of ssDNA into empty capsids (Fig. 2h) (Cotmore and Tattersall, 1987; Willwand and Kaaden, 1990). NS1 is a cytotoxic protein that can induce apoptosis, which is the hallmark of productive AMDV infection (Fig. 2i) (Leimann et al., 2015; Moffatt et al., 1996). Apoptosis releases the matured virion and cell antigens inside the infected cells (Fig. 2j). Steps a, b, d, and g are partially hypothetical (Qiu et al., 2017).

Subsequent to the infection with AMDV, the disease manifests differently depending on the strain and dose of the virus and the host genotype and age (Canuti et al., 2015; Farid and Hussain, 2020). Moreover, the virus possesses different pathology and cell-tropism in fetuses, kits, and adults (Best and Bloom, 2006, 2005). Transplacental transmission of AMDV occurs from both persistently and acutely infected dams, resulting in abortion, absorption of the fetus, or embryonic death. The virus can permissively replicate in fetal tissues(Best and Bloom, 2006, 2005). AMDV was found in different fetal tissues, including the liver, spleen, connective tissue of the skin, interstitial connective tissue, heart muscle cells, thymus, bone marrow, lung, brain, and placenta(Broll and Alexandersen, 1996). AMDV could infect immature hematopoietic cells and hepatocytes in liver of fetuses; however, there is limited knowledge of the infected cell types in other fetal tissues (Broll and Alexandersen, 1996).

**Fig. 3:**
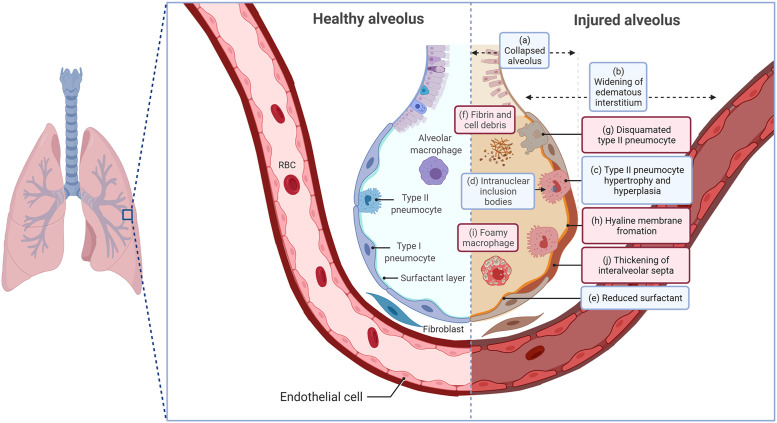
Pathologic changes in acute interstitial pneumonia caused by Aleutian mink disease virus in mink kits.

The acute form of AD with fatal respiratory distress and fulminant interstitial pneumonia mainly occurs in kits, which is due to permissive and cytopathic replication of the virus in the lung type II pneumocytes and the subsequent impaired surfactant production (Alexandersen, 1986; Bloom et al., 1994). The pathogenesis of AMDV in kits’ lung tissue is briefly depicted in Fig. 3, primarily based on the data gathered by Alexandersen's studies (Alexandersen, 1986; Alexandersen et al., 1987; Alexandersen et al., 1994a, 1994b; Alexandersen and Bloom, 1987). Pathologic changes of early stages (Fig. 3, blue boxes) consist of collapsed pulmonary alveoli (Fig. 3a), interstitial edema (Fig. 3b), hyperplasia of type II pneumocytes (Fig. 3c), intranuclear inclusion bodies in type II pneumocytes (Fig. 3d), and decreased surfactant production (Fig. 3e). Pathologic changes of later stages (Fig. 3, red boxes), include fibrin and cellular debris deposition (Fig. 3f), accumulation of desquamated type II pneumocytes (Fig. 3g), hyaline membrane formation (Fig. 3h), overpopulation of alveolar macrophages (Fig. 3i), and diffuse thickening of interalveolar septa (Fig. 3j). Survived kits also have hypertrophy of bronchus-associated lymphoid tissue and focal subpleural, intraalveolar accumulations of large cells with foamy cytoplasm, so-called “lipid pneumonia” (Alexandersen et al., 1994a).

In adults, a persistent chronic infection caused by the virus results in progressive wasting syndrome (Eklund et al., 1968; Hadlow et al., 1984). It has been suggested that the viral-induced cell cycle arrest makes the infected cells poor targets for cytotoxic T lymphocytes (CTLs), which promotes the intracellular persistence of AMDV (Oleksiewicz and Alexandersen, 1997). In this form of AD, the virus noncytopathicly replicates in lymphoid tissue, specifically macrophages and B cells; however, the replication of the virus is partially restricted with cytotoxic T cells (Alexandersen et al., 1988; Jensen et al., 2000; Kanno et al., 1992). The humoral immune response plays a pivotal role in the pathogenesis of the virus in adults, as a severe polyclonal hypergammaglobulinemia or plasmacytosis, with γ-globulin constituting up to half of the total serum proteins, is the hallmark of the progressive form of AD (Bloom et al., 1994). The progressive increase in serum immunoglobulin (Ig) in AMDV-infected mink results from a specific antiviral response and an autoimmune response (Aasted et al., 1984). A large portion of autoimmune antibody is due to anti-DNA antibody, which is more strongly associated with gamma globulin levels than antiviral antibody (Hahn and Hahn, 1983). IgG is the primary elevated Ig; although, a transient but significant increase in serum IgM levels can be detected in the early stages of infection (Porter et al., 1984).

Antiviral antibodies can be neutral, protective, or fatal based on the host's age. Antiviral antibody plays neither protective nor pathogenic roles in fetuses (Best and Bloom, 2005). In contrast, in kits, the development of severe acute disease is associated with low or absent antibody titers paired with high levels of permissive viral replication (Alexandersen et al., 1989). However, the passive transmission of anti-AMDV antibodies (IgM) can restrict viral replication and transcription, reduces both mortality and severity and protects kits during the period in which animals are susceptible to acute disease (Alexandersen et al., 1989). Antibodies against AMDV enhance the entry of the virus into adult mink's macrophages, which is mediated by cellular Fc receptors (FcR), through antibody-dependent enhancement (ADE) process (Dworak et al., 1997; Kanno et al., 1993). Antibodies against the VP2:428–446 residue mediate the ADE and aggregation of virus particles into immune complexes (Bloom et al., 2001).

**Fig. 4:**
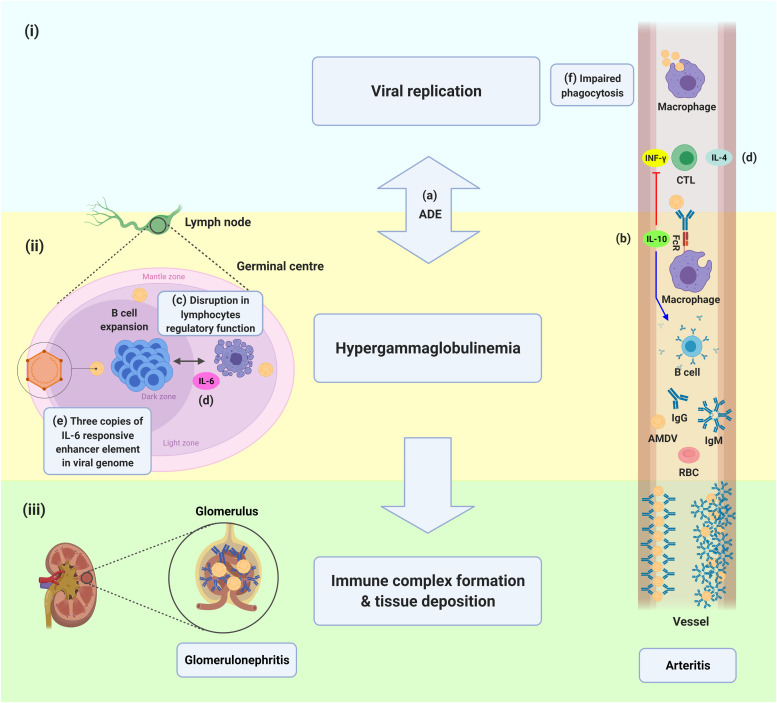
The Aleutian mink disease virus and mink immune system interactions in the progressive form of disease.

Fig. 4 provides a brief overview of AMDV and host immune system interaction in an adult mink with progressive AD. There is a strong linkage between viral replication (Fig. 4i) and hypergammaglobulinemia (Fig. 4ii) since the antiviral antibodies cannot eliminate the virus and, reversely, promote the viral entrance to the host cells and boost viral replication (Kanno et al., 1993). In contrast, virus replication is associated with more production of antibodies. Various mechanisms have been suggested for AMDV-induced hypergammaglobulinemia. Antibodies enhance the entry of the virus into adult mink macrophages, known as ADE process (Fig. 4a) (Kanno et al., 1993). This defective cycle of immune enhancement, in which binding the virus to the non-neutralizing antibodies enhances its entry into macrophage cells, multiplies the final consequence of viral entrance and replication, in this case, antibody production (Kanno et al., 1992). The ligation of FcR (Fig. 4b) resulted in the production of interleukin (IL)−10, which provokes antibody production and suppression of interferon signaling pathway induced by CTL responses, two essential restriction elements of virus persistence and replication (Best and Bloom, 2005). Another explanation is the impaired down-regulation of the germinal center reaction by tingible body macrophages (Fig. 4c), resulting in unrestricted expansion of B cell populations and antibody production (Jensen et al., 2000). The upregulation of cytokines of IL-4, mainly in CTLs, and IL-6 in infected macrophages stimulate differentiation of B cells into plasma cells, enhance antibody production, and facilitates virus replication (Fig. 4d) (Jensen et al., 2003). Upregulation of IL-4 and IL-6 is consistent with the development of plasmacytosis (Jensen et al., 2003). The AMDV genome also includes three copies of a sequence identical to the consensus sequence for an IL-6- responsive enhancer element, which may exacerbate plasmacytosis (Fig. 4e) (Donkor and Farid, 2008). The mononuclear phagocytic system blockade and the consequence of impaired phagocytosis in infected mink may be responsible for the pathogenicity of the virus to some extent (Fig. 4f) (Lodmell et al., 1990). The antiviral antibodies produced account for the majority of AD clinical manifestation. Since anti-AMDV antibodies could bind to the virus but cannot effectively neutralize it, the virus-antibody complexes may deposit in the blood vessels and renal glomeruli, leading to arteritis and glomerulonephritis (Fig. 4iii) (Cheema et al., 1972).

### 3.1 Virus strains and mink genotypes

AMDV strains are divided into three groups: (i) highly pathogenic strains, including AMDV-Utah 1, -TR, -United, and -K; (ii) non-pathogenic strain of AMDVG; (iii) other isolates of AMDV exhibiting intermediate pathogenicity (Bloom et al., 1998; Gottschalck et al., 1994). Highly virulent isolates can cause severe disease in adult and newborn animals, with mortality rates approaching 100% in Aleutian and non-Aleutian genotypes of mink (Aleutian color) (Alexandersen et al., 1994a; Oie et al., 1996). In contrast, AMDVG does not induce AD in adult mink and has lesser pathogenicity for kits (Alexandersen et al., 1994b; Bloom et al., 1990, 1980). Pathogenic strains replicate faster, develop earlier symptoms, and induce more severe pathologic lesions than minor pathogenic strains, and this might result from higher antiviral antibody production (Aasted et al., 1984; Oie et al., 1996; Stevenson et al., 2001). The VP2 hypervariable coding region and valine residue at codon 352 in the VP2 capsid protein have been also suggested as virus pathogenicity determinants (Oie et al., 1996; Stevenson et al., 2001).

Aleutian mink is susceptible to almost all AMDV strains except for the non-pathogenic AMDVG strain. In contrast, non-Aleutian mink are variably susceptible to the infection, which can be mainly categorized into two general forms: (i) typical progressive disease with hyperglobulinemia and histopathologic lesions; (ii) persistent non-progressive infection with slight increases of gamma globulin without lesions (Best and Bloom, 2005). There is inconsistency in the presence of another form of disease known as “non-persistent, non-progressive infection with the clearance of the virus”. Previous literature reported this type of immune response in non-Aleutian mink infected by Pastel and Pullman viral strains (Hadlow et al., 1984; Larsen and Porter, 1975), while no recent evidence for viral clearance exists. More recently, Jensen et al. (Jensen et al., 2014) demonstrated that in chronic infection of Sapphire mink with a field strain of AMDV, all animals remained seropositive throughout the 24 weeks of the study, and no viral clearance was identified.

The persistent non-progressive infection can be generated as a result of: (i) higher T helper type 1 activity leading to higher production of IFN-producing cells and development of high specific CTLs; (ii) antibody response to the correct antigen or antigens of the virus, (iii) restricted viral replication and antibody production at low levels in response to the sequestered virus, and (iv) the absence of cross-reactive antigens from other proteins which may cause persistent antibody production (Best and Bloom, 2005; Jensen et al., 2003). Therefore, animals with high coordination between cellular and humoral immune responses are more likely to develop less severe hypergammaglobulinemia. This disparity implicates different host factors as the primary source of variation in the kinetics of antibody production and provides the opportunity to select the high-coordinated immune response for disease control.

Studies on different color types of mink found various responses against AMDV. In a study performed by Hadlow et al. (Hadlow et al., 1983) a higher rate of mortality post-inoculation was detected in sapphire than pastel mink. Moreover, sapphire mink showed higher levels of antibody production post-inoculation than pastel genotype (Bloom et al., 1975). Another study by Lodmell et al. (Lodmell et al., 1973) demonstrated that sapphire mink are more susceptible than pastel to the Pullman isolate of AMDV. Since most of the studies on the differences in response to AMDV among different mink colors were performed in the 1970s and 1980s, further investigation of the differences in response to AMDV among different mink genotypes could be helpful for mink breeding programs.

### 3.2 Viral dose

Low doses of the virus cause variable responses among mink, while high doses generally can overwhelm the host's innate immune defense in almost all animals and result in infection (Farid and Hussain, 2020; Hadlow et al., 1983). Differences in viremia and antibody production among individuals can primarily be identified in infections with low doses of the virus, which naturally occurs in mink farms (Farid and Hussain, 2020). Interestingly, this might enhance the chance of successful mink selection programs for AD tolerance in order to control the disease. When the host is exposed to high doses of the virus, a higher probability of the incidence of viremia and hypergammaglobulinemia is expected (Farid and Hussain, 2020).

## 4 Clinical manifestation of AD and its impacts on animal's performance

The majority of clinical signs develop within eight weeks after infection (Jensen et al., 2015). In kits, an infection with highly virulent strains results in an incidence and mortality of > 90% (Alexandersen, 1986). In contrast, low virulent strains cause a 50–70% incidence rate and 30–50% mortality (Alexandersen, 1986). The kits’ respiratory distress syndrome is the most frequent reason for death post-AD infection (Best and Bloom, 2005). However, the kits that survived the acute infection will develop typical lesions of the classical adult form of AD, regardless of the virus strain (Alexandersen, 1986). The disease in adults is characterized by weight loss, anorexia, lethargy, roughened coat, hair depigmentation, pale mucous membranes, coma, and death (Eklund et al., 1968; Farid and Hussain, 2020; Hadlow et al., 1984). Aleutian disease progression can affect the reproductive performance of females; this means the risk of infertility among mink with high levels of anti-VP2 antibody before mating is more than those with low antibody levels (Andersson et al., 2017). If the infection occurs before the mating, although the female already has high antibody titers, the virus can cross the endotheliochorial placental barrier and increases the incidence of abortion and resorbed fetuses (Broll and Alexandersen, 1996). Moreover, smaller litter sizes, lower litter weights, and increased neonatal mortalities are expected (Andersson et al., 2017; Reichert and Kostro, 2014). The disease also depresses pelt market value by developing white hair called “sprinklers” (Farid and Ferns, 2011). Therefore, selection for female mink with disease tolerance not only helps to control AD and decreases the risk of having barren females and early kit mortality but also can improve litter size, litter weight, and pelt quality.

## 5 Diagnosis and disease progression estimation

AD diagnosis is primarily based on detecting antiviral antibodies or viral antigens. The diagnostic tools can be classified into: (i) non-specific and specific immunoassays, (ii) AMDV molecular detection tests, and (iii) quantitative assessment of total gamma globulin or anti-AMDV antibody levels. During the late 1960s and early 1970s, several non-specific assays, including serum electrophoresis (Henson et al., 1961), iodine agglutination test or IAT (Henson et al., 1962), and glutaraldehyde test (Sandholm and Kangas, 1973) were developed to evaluate the levels of serum globulin. Regardless of the source of infection, these tests would achieve positive results when high levels of serum globulin are detected, representing their low specificity. However, the contemporary AMDV eradication strategies are inspired by the application of IAT in the early 1960s. During that time, mink with high globulin levels (gamma globulin > 2 g/10 ml of serum or albumin-to-globulin ratio > 1) were considered as positive animals and were culled (Farid et al., 2018; Gorham et al., 1965).

### 5.1 Non-specific and specific immunoassays

In 1972, a counter-immunoelectrophoresis (CIEP) test to detect either AMDV antigen or anti-AMDV antibody was established as a rapid, sensitive serologic method (Cho and Ingram, 1972). Principally, the test is based on the visual detection of precipitin lines resulting from the immune complex formation on an agarose gel after electrophoresis. Before the availability of the *in vitro*-grown antigen of AMDVG in the 1980s, the assay was carried out with viral antigen extracted from tissues such as spleen, liver, and kidney of infected animals (Aasted and Cohn, 1982). The CIEP assay has been widely used for routine AD diagnosis and eradication programs in Canada and Denmark. Subsequent to CIEP, several immunoelectrophoretic assays have been developed to increase its sensitivity and specificity, including modified counterelectrophoresis (Crawford et al., 1977), inhibition of precipitation in counter-current electrophoresis (Aasted and Cohn, 1982), rocket line immunoelectrophoresis (Alexandersen and Hau, 1985), counter-current line absorption immunoelectrophoresis or CCLAIE (Aasted et al., 1986), thin‐layer CCLAIE (TL‐CCLAIE) (Alexandersen et al., 1985), and additive counterimmunoelectrophoresis (Uttenthal, 1992). However, none of these assays could replace the original CIEP due to their high costs, time-consuming, or laborious processes. The CIEP test is known as the gold standard of AD diagnosis due to its high specificity and reasonable sensitivity, although the test is unsuitable for high-throughput screening because of the time-consuming process and dependency on large quantities of antigen (Ma et al., 2016). Moreover, CIEP is not recommended for eradication programs as seropositive animals may remain in the herd due to its relatively low sensitivity, which could result in an uncontrollable spread of the disease. Additionally, the results of CIEP are subjective, i.e., reading the test demands experience, which leads to higher false-positive/false-negative outcomes (Ma et al., 2016). These reasons for the incompetence of CIEP may explain Canada and Denmark AD eradication programs failure. However, low biosecurity level (Compo et al., 2017; Prieto et al., 2018; Themudo et al., 2012), interchange of virus between wild and farmed animals (Gunnarsson, 2001; Oie et al., 1996), and persistence of the virus in the environment (Larsen, 2013; Prieto et al., 2014) were proposed as other causal elements of the failures.

Currently, enzyme-linked immunosorbent assay (ELISA) is the most common method for routine screening of AD. However, the results from the first developed ELISAs using AMDVG antigen were not satisfactory and showed very low sensitivity than CIEP (Andersson and Wallgren, 2013). With the availability of recombinant VP2 protein, the opportunity to develop more sensitive ELISA tests was provided (Clemens et al., 1992). Table 1 represents the specificity and sensitivity among different AD serologic tests. The first ELISA method to detect anti-VP2 antibodies was developed by Knuuttila et al. (Knuuttila et al., 2009a). The test was further improved to an automated high-throughput ELISA system using blood samples collected by filter paper strips, which facilitated blood sampling and reduced test time, costs, and labor intensity, while maintaining high sensitivity and specificity (Knuuttila et al., 2014). Another automated ELISA method using AMDVG antigen was also designed to screen Danish mink farms that showed high sensitivity and specificity (Dam-Tuxen et al., 2014). Furthermore, two ELISA systems based on VP2332-452 and P1 peptide have been described in China, with specificity and sensitivity of more than 97% (Chen et al., 2016; Ma et al., 2016).

**Table 1:** Sensitivity and specificity of Aleutian disease serological tests applied in eradication programs worldwide.

Method	Country	Antigen	Sensitivity	Specificity	Reference(s)
CIEP	Canada	AMDVG	72.9–100%	99.8–100%	(Cho and Ingram, 1972; Dam-Tuxen et al., 2014; Knuuttila et al., 2009a)
AMDVG ELISA	USA	AMDVG	54.3%	93.2%	(Andersson and Wallgren, 2013)
VP2 ELISA	Finland	Finish wild-type recombinant VP2 protein	99%	97%	(Knuuttila et al., 2009a)
High-throughput automated VP2 ELISA	Finland	Finish wild-type recombinant VP2 protein	96.2%	98.4%	(Knuuttila et al., 2014)
High-throughput automated AMDVG ELISA	Denmark	AMDVG	72.6–93.1%	98.8–100%	(Dam-Tuxen et al., 2014)
VP2_332–452_ peptide ELISA	China	VP2_332–452_ recombinant peptide	97.3%	97.9%	(Chen et al., 2016)
P1 peptide ELISA	China	P1 recombinant peptide	98.0%	97.5%	(Ma et al., 2016)

### 5.2 Molecular detection

CIEP test cannot detect low levels of antibodies during the early stages of infection; however, polymerase chain reaction (PCR) can accurately identify the virus in blood and lymphoid organs during the initial stages of infection (Farid et al., 2015). Moreover, the sensitivity of CIEP at 20 days post-infection is considerably lower than PCR (Farid and Hussain, 2020). Even though, a single PCR test may not be sufficient to detect AMDV accurately, and multiple tests are needed to increase the chance of detection (Farid, 2013; Farid and Ferns, 2017). The type of sample is a vital factor in molecular detection of AMDV, as short-lived viremia in chronically infected animals leads to poor detection of virus when blood samples are used (Farid and Hussain, 2020, 2019). Other samples, such as saliva, stool, and urine, are unsuitable for detecting infection due to their low sensitivity, the difficulty of sample collection, and the risk of contamination (Farid et al., 2015; Farid and Hussain, 2020; Jensen et al., 2014).

### 5.3 Quantitative analysis of total antibody or anti-AMDV antibody levels

With the failure of eradication strategies, more attention was given to building up AD-tolerant herds through selection programs. Similar strategies have been applied to produce resistant lines of chickens to Marek's disease and avian leukosis (Bacon et al., 2000). Most AMDV field strains have low pathogenicity leading to a sufficient number of animals with tolerant phenotypes, which provides the selection opportunity (Henson et al., 1976). Detecting the virus or antiviral antibody is not practical, sensitive, and specific for determining tolerant animals (Hadlow et al., 1983). Hence, monitoring the disease progression by estimating the level of hypergammaglobulinemia or anti-AMDV antibody during the disease is inevitable in selection programs for AD tolerance and controlling the disease.

Quantitative assays can be classified into two groups, including tests: (i) measuring serum gamma globulin levels and (ii) measuring anti-AMDV antibody levels. Tests of the first group are non-specific, meaning any factor increasing the animal's total antibody can confound the results. The IAT, the most common among the first group tests, has been used in some regions of North America and Europe to select tolerant mink. However, the failure of the IAT-based selection strategies could be connected with its low specificity and inability to detect low gamma globulin levels in non-progressive forms of AD (Farid et al., 2018). Another measurement classified in the first group is the albumin to globulin ratios (A:γG) using conventional electrophoresis of serum proteins and matrix-assisted laser desorption/ionization-time-of-flight or MALDI-TOF (Cepica et al., 2012; Henson et al., 1966). Similarly, A:γG ratio is also non-specific, laborious, time-consuming, expensive, and unsuitable for high throughput testing.

**Fig. 5:**
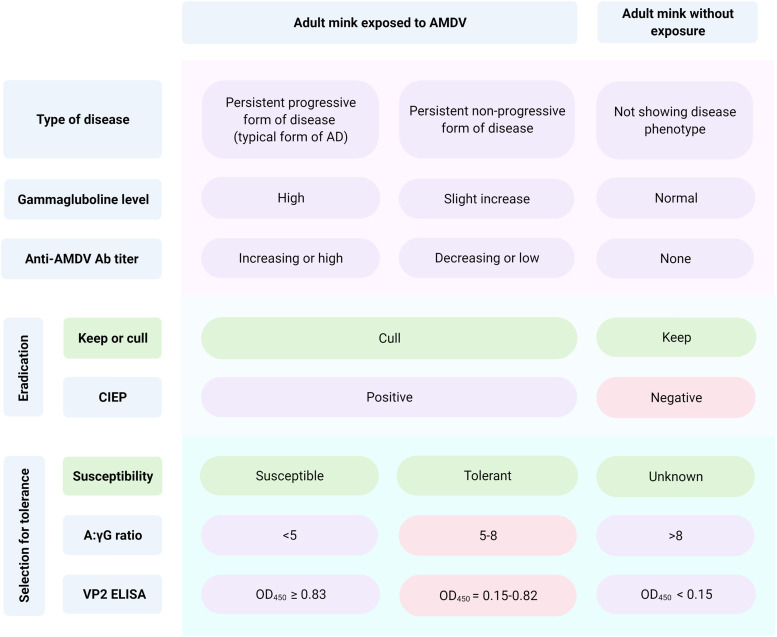
Application of different Aleutian disease serological assays regarding the applied strategy. The red boxes show the desired result of the tests in each approach. Values for albumin to globulin ratios (A:γG) and VP2 ELISA are based on Cepica et al. (2012) and Andersson et al. (2016) studies, respectively.

Currently, it is feasible to estimate the anti-AMDV antibody levels using quantitative ELISA systems (Farid and Rupasinghe, 2016). Aleutian disease quantitative ELISAs can be classified based on the antigen utilized to detect antibody levels: (i) AMDVG ELISA; and (ii) VP2 ELISA. However, one study showed that measurements using VP2 antigen have higher accuracies than AMDVG antigen (Farid and Rupasinghe, 2016). Moreover, between VP2 ELISA and high-throughput automated VP2 ELISA, the latter has been found a promising tool for estimation of AD progression (Andersson et al., 2015). Therefore, infected mink with low antiviral antibody levels could be of interest to select for AD tolerance (Andersson et al., 2016). Fig. 5 provides information on the application of described tests in two different types of breeding strategies against AD, including eradication or selection for AD tolerance programs.

## 6 AD control

Aleutian disease control is important both economically and from the aspect of animal welfare. Among the countries that applied eradication strategies, elimination of AD was only achieved in Iceland for twelve years (1984–1996) using intensive CIEP testing, elimination and disinfection of positive mink ranches, and repopulation of farms (Gunnarsson, 2001). The eradication strategies in other regions, such as Canada (Nova Scotia), failed due to the dense mink breeding and virus transmission between farm and wild animals. Therefore, with the absence of an effective treatment or completely protective vaccine, the significance of AD control by applying strict biosecurity, control of biologic vectors, monitoring of wild mutilids infections, and precise breeding programs for AD tolerance is increased.

Various wild mutilids, e.g., wild mink, short-tailed weasels, foxes, lynx, American martens, North American river otters, and striped skunks, can be the wild reservoirs of AMDV (Canuti et al., 2020; Farid, 2013; Farid et al., 2010; Knuuttila et al., 2015; Manas et al., 2001; Nituch et al., 2011). The recent rapid expansion of Amdoparvovirus genus infections to different wild mustelids suggests that wild animals can be a threat to farmed mink in the case of increased control of AMDV (Canuti et al., 2020). The high seroprevalence of AD in various wild mutilids unsealed the critical role of feral animals as a possible source of infection or re-infection of clean mink ranches. An explanation for the failure of eradication strategies could be infected feral animals that are in direct or indirect contact with farmed mink. Reversely, infected farms also may play an important role in dispersing AMDV in a region. A study by Canuti et al. (2020) showed a higher prevalence of AD in mink harvested near AMDV-affected fur farms, and their viruses were phylogenetically closely related to those from farms. Effective security fences, self-closing, lockable gates, and enclosed sheds to minimize wildlife access to mink can eliminate the contact between farmed and wild animals and reduce the chance of AMDV transmission (Compo et al., 2017).

### 6.1 Application of genomic selection for AD control

**Fig. 6:**
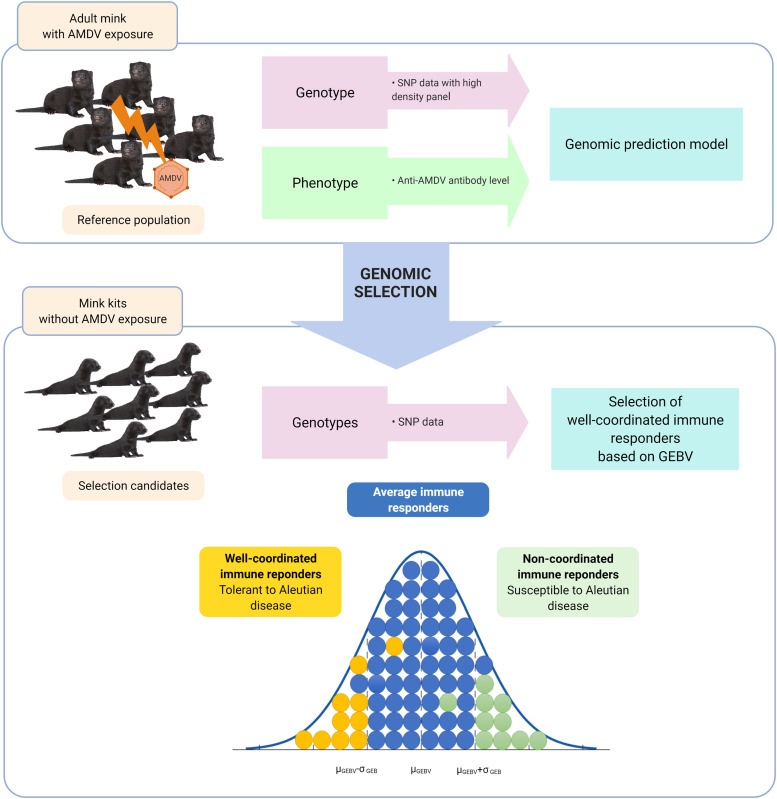
Application of genomic selection to build tolerant mink herds. In genomic selection, a reference population, including individuals with phenotypic records and genotypes, is used to develop a genomic prediction model. This model will be later used to predict the genomic estimated breeding values (GEBVs) of genotyped young animals to select superior animals to contribute to the next generations.

Traditional genetic improvement of livestock has been quite successful in predicting breeding values using phenotypic and pedigrees data. However, breeding values are able to predict the next generations more accurately using information on variations in DNA sequence between animals. Genome-wide single nucleotide polymorphisms (SNP) data, commonly used as the primary source of genomic information, provides a great opportunity for estimation of more accurate genomic breeding values (GEBV) for individuals compared to traditional approaches (Meuwissen et al., 2016). In this approach, known as “genomic selection”, once each SNP effect is found by combining animals’ genotypes with the estimated breeding values (EBVs), GEBVs are calculated for the selected candidates (Georges et al., 2019). Genomic selection has been found as the superior methodology of genetic evaluation in livestock breeding programs, especially for disease-linked traits, since pedigree recording is not necessarily required (Hayes et al., 2013). Quantitative traits with low-to-moderate heritability, such as disease tolerance, are largely affected by environmental effects; therefore, accurate prediction of them is very challenging. However, genomic selection has been more effective for these traits since it uses genomic marker information to predict the GEBVs in the testing population (Hayes et al., 2013). Using genomic selection, accurate estimates of genetic merit can be achieved by exploiting the animal's genotype in the earliest stages of the animal's life without phenotypic information about the disease (Hayes et al., 2013). Other benefits of genomic selection include increasing rates of genetic gain, minimizing inbreeding, and limiting potential effects of genotype by environment interactions (Hayes et al., 2013). Fig. 6 schematically depicts the hypothetical application of genomic selection to build tolerant mink herds for AD control.

Several quantitative approaches have been proposed to investigate the immune response variations among individuals and classification of animals based on their immune response. In the immunocompetence approach, animals are ranked based on the antibody and cellular immune responses against non-pathogenic antigens, e.g., vaccines (Hine et al., 2019; Wagter-Lesperance and Mallard, 2007). This method can classify the animals as high, average, or low responders based on their EBVs or GEBVs (Mallard et al., 2015). It has been claimed that high responders have the inborn ability to mount balanced and effective immune responses compared with average or low responders (Mallard et al., 2015). However, it is noteworthy that in most infectious diseases, the high immune responders are preferably selected for immune traits in breeding programs (Detilleux et al., 1995; Thompson-Crispi et al., 2013). But, in the case of AD, exposed mink with higher cellular and humoral immune response coordination against the virus are preferred, which are characterized by lower anti-AMDV antibody levels in traditional phenotypic selection or, more precisely, by lower GEBVs in genomic selection approach. Therefore, it is feasible to classify animals based on their GEBVs estimated for anti-AMDV antibody levels into well-coordinated, average, and non-coordinated immune responders (Fig. 6).

The immunocompetence approach provides an opportunity to select animals which are tolerant to different pathogens, although differences in their pathogenicity may restrict its power to select animals for tolerance to multiple pathogens. With the recent discovery of severe acute respiratory syndrome coronavirus 2 (SARS‐CoV‐2)-infected mink farms in different countries, such as Denmark, the Netherlands, Italy, Greece, Spain, and the USA, breeders may favor an immunocompetence approach to build multi pathogen-tolerant herds (Hammer et al., 2020; Koopmans, 2021; Opriessnig and Huang, 2020; Oreshkova et al., 2020). Hence, more studies are necessary to investigate the genetic correlations of the preferred immune response traits against various pathogens.

Defining the relevant phenotypes as AD tolerance and susceptibility seems to be the main challenge of discovering the genetic footprints of AD in the single-disease strategy. Mink tolerant to AD can cope with the presence of the virus by maintaining their production values, while experiencing slight hypergammaglobulinemia post-infection. However, a single measure of AMDV antibodies titer may not accurately identify the tolerant animals due to the differences in the time of the infection establishment (Andersson et al., 2016). Tolerant and susceptible animals could be found in populations using multiple ELISA tests, preferably VP2-based ones. The CIEP test alone is not specific for distinguishing the susceptible from the tolerant (Fig. 5).

### 6.2 Challenges and potentials for the implication of genomic selection programs for AD tolerance

The first question that would arise is if AD-resistant animals exist, and in case of their presence, is selection for AD resistance preferred over its tolerance. There is insufficient evidence of existing AD-resistant animals that can clear the virus through an effective immune response. In these animals, the host-pathogen interactions lead to co-evolution of antagonistic traits in host and virus, i.e., if a host is selected for resistance to a virus, the microorganism will evolve a method to subvert the resistance. Consequently, selection for AD-resistant mink might be in concert with a simultaneous selection pressure for escaping from resistance mechanisms in the virus. This co-evolutionary relationship prevents the resistance trait from becoming fixed within a host population (Schneider and Ayres, 2008). Considering the fact that parvoviruses such as AMDV have a high mutation rate, with 10^−6^ to 10^−4^ substitutions per nucleotide site per cell infection (Sanjuán et al., 2010), the evolution process of the virus may occur after a few hosts generations, eventually, leads to the failure of breeding programs for AD resistance.

In contrast, tolerance has a neutral or possibly positive effect on the pathogen because tolerant animals live longer by alleviating infection severity, thereby enhancing the prevalence of the disease and potentially altering its spread. Conversely, a tolerance trait will eventually become fixed in a host population because it will be positively selected. Mechanisms that increase tolerance are not predicted to result in the evolution of highly resistant pathogens (Schneider and Ayres, 2008). Moreover, selection for tolerance may create a cross-protection against different virus strains or other infectious agents (Ayres and Schneider, 2012). Hence, selection for AD tolerance could be more advantageous and lasting. Commercial mink farming experienced rapid changes over the last few years due to the risk of spreading zoonotic viruses such as SARS-CoV-2 or avian influenza A virus (IAV) H5N1 (Agüero et al., 2023; Oude Munnink et al., 2021). This has resulted in the closure of many mink farms, especially in Europe, which may serve to reduce the genetic diversity of AMDV ultimately. For instance, human infection with variant mink viruses with spike mutations led to the culling of all mink in Denmark (Hammer et al., 2020; Larsen et al., 2021). Therefore, it is necessary to investigate what are the genetic correlations among tolerance against AMDV, SARS-CoV-2, and IAV-H5N1 and if higher levels of SARS-CoV-2 or IAV-H5N1 virus replication are observed in AMDV-infected mink.

An additional challenge of selection programs for AD tolerance could be the lower heritability of disease tolerance traits than immune response traits; consequently, the genetic gain will be slower (Emam et al., 2019; Xu et al., 2017). In contrast, Bishop and Woolliams's theory (Bishop and Woolliams, 2010) propose that traits describing components of immune responses to infection, e.g., antibody production, are often highly heritable. In case of AD, there is an opportunity to select tolerant animals based on their antibody levels against AMDV. The heritability of antibody response against AMDV using the two ELISA platforms of AMDVG and VP2 were previously reported as 0.39±0.06 and 0.61±0.07 (Hu et al., 2021); therefore, ELISA tests, particularly VP2 ELISA, have the potential to be an indicator for genetic or genomic selection of AD tolerant mink.

Negative genetic correlations of tolerance to other pathogens or significant production or reproduction traits could be challenging in breeding programs. Aleutian disease reduces fertility rates and pelt value in infected herds. Therefore, it is expected that tolerant animals have higher reproductive and productive performance, which could be due to the positive genetic correlation between these traits and AD tolerance. It is necessary to define breeding program goals by considering the production and reproduction traits of interest and epidemiologic data on common pathogens of that location. Therefore, in applying a single-disease approach to build AD-tolerant mink farms, genetic correlations with immune response traits to other local diseases, production and reproductive traits, and the availability of accurate AD tests to exploit tolerant animals should be taken into account in establishing breeding goals. Indeed, the positive impacts of AD tolerance selection on production traits may guarantee the genomic improvement programs for AD tolerance.

The quality of response against infectious agents in newborns is mainly due to maternal antibodies. However, whether AD-tolerant dams can reproduce more tolerant kits through trans-placental antibody transfer and colostrum is still unknown. No information exists regarding the effects of AD tolerance selection on the quality of immune response to other infectious agents and vaccines. Moreover, the cost-effectiveness of genomic selection for AD tolerance in mink should be evaluated as it is not well known to what extent genomic selection can help reduce AD seroprevalence in mink populations. More studies are necessary on the host-pathogen-environment interactions of AD. The results of these studies would help better design the breeding programs and guarantee AD control.

In conclusion, AD is a serious infectious disease with devastating consequences for the mink industry. We discussed the importance of AD in the mink industry, the pathogenicity of ADMV, the availability of serological and molecular tests, the best options for monitoring AD progression in infected animals, and how these tests can be applied to selection programs. With the availability of quantitative ELISA systems and cost-effective high throughput genomic data, we can precisely estimate the genomic merit of animals for AD tolerance. Meanwhile, more studies are necessary to understand the genetic correlation of AD tolerance with productive and reproductive traits as well as host response against other important pathogens. With genomic selection, breeders can decrease the breeding cycle time, increase selection intensity, and boost the overall rate of genetic gain for AD tolerance. Aleutian disease tolerance needs to be one of the key traits predicted for selection in the mink industry.

## Funding statement

This research did not receive any specific grant from funding agencies in the public, commercial, or not-for-profit sectors.

## CRediT authorship contribution statement

**Seyed Milad Vahedi:** Conceptualization, Methodology, Visualization, Writing – original draft. **Siavash Salek Ardestani:** Visualization, Writing – review & editing. **Mohammad Hossein Banabazi:** Writing – review & editing. **Fraser Clark:** Writing – review & editing.

## Declaration of Competing Interest

The authors declare that they have no conflict of interest.

## Data Availability

No data was used for the research described in the article.
